# Cost-effectiveness of transcatheter aortic valve implantation in patients with severe symptomatic aortic stenosis of intermediate surgical risk in Singapore

**DOI:** 10.1186/s12913-022-08369-5

**Published:** 2022-08-04

**Authors:** Rachel Su-En See-Toh, Xin Yi Wong, Kush Shiv Kishore Herkshin Mahboobani, Swee Sung Soon, Benjamin Kearns, Katy Cooper, Kay Woon Ho, Ivandito Kuntjoro, Kwong Ng

**Affiliations:** 1 Agency for Care Effectiveness, Ministry of Health, Singapore, Singapore; 2 School of Health and Related Research, University of Sheffield, Sheffield, UK; 3 National Heart Centre, Singapore, Singapore; 4 National University Heart Centre, Singapore, Singapore

**Keywords:** Transcatheter aortic valve implantation, Transcatheter aortic valve replacement, TAVI, TAVR, Cost-effectiveness, Singapore

## Abstract

**Objective:**

The objective was to assess the cost-effectiveness of transcatheter aortic valve implantation (TAVI) in patients with severe aortic stenosis with intermediate surgical risk in Singapore.

**Methods:**

A de novo Markov model with three health states – stroke with long-term sequelae, no stroke, and death – was developed and simulated using Monte Carlo simulations with 10,000 iterations over a five-year time horizon from the Singapore healthcare system perspective. A 3% annual discount rate for costs and outcomes and monthly cycle lengths were used. By applying the longest available published clinical evidence, simulated patients received either TAVI or surgical aortic valve replacement (SAVR) and were at risk of adverse events (AEs) such as moderate-to-severe paravalvular aortic regurgitation (PAR).

**Results:**

When five-year PARTNER 2A data was applied, base-case analyses showed that the incremental cost-effectiveness ratio (ICER) for TAVI compared to SAVR was US$315,760 per quality-adjusted life year (QALY) gained. The high ICER was due to high incremental implantation and procedure costs of TAVI compared to SAVR, and marginal improvement of 0.10 QALYs as simulated mortality of TAVI exceeded SAVR at 3.75 years post-implantation. One-way sensitivity analysis showed that the ICERs were most sensitive to cost of PAR, utility values of SAVR patients, and cost of TAVI and SAVR implants and procedures. When disutilities for AEs were additionally applied, the ICER decreased to US$300,070 per QALY gained. TAVI was dominated by SAVR when the time horizon increased to 20 years. Clinical outcomes projected from one-year PARTNER S3i data further reduced the ICER to US$86,337 per QALY gained for TAVI, assuming early all-cause mortality benefits from TAVI continued to persist. This assumption was undermined when longer term data showed that TAVI’s early mortality benefits diminished at five years.

**Limitations and conclusion:**

TAVI is unlikely to be cost-effective in intermediate surgical-risk patients compared to SAVR in Singapore.

**Supplementary Information:**

The online version contains supplementary material available at 10.1186/s12913-022-08369-5.

## Introduction

Aortic stenosis refers to the narrowing of the aortic valve, which obstructs blood outflow from the left ventricle to the aorta [1].In Singapore, the estimated prevalence of aortic stenosis increased from 3% in adults older than 75 years to about 8% in those above 85 years [2]. Patients with symptomatic severe aortic stenosis who did not undergo aortic valve replacement (AVR) experienced high mortality [3, 4]. About 44% of patients with symptomatic severe aortic stenosis died within a median follow-up of 14.5 months [5].Transcatheter aortic valve implantation or replacement (TAVI or TAVR) is a minimally invasive procedure that replaces the stenosed valve with a catheter-deployed bioprosthetic valve, often considered as an alternative to conventional surgical aortic valve replacements (SAVR) [2, 6]. The expansion of regulatory approval for lower surgical risk groups has led to its increased uptake, catalysed by its less invasive nature, and improved technology, patient selection and operator learning curve over time [7–9]. Published evidence in intermediate surgical risk patients suggested that clinical effectiveness outcomes for TAVI were comparable to SAVR in randomised controlled trials (RCTs) and a propensity-score matched study (PSM). The study designs of published evidence varied with respect to the length of follow-up and the generation of TAVI device i.e. RCTs had longer follow-up with an earlier generation TAVI device while the PSM had shorter follow-up with later generation TAVI [10–14]. Unlike the published evidence for patients with aortic stenosis who were deemed to be inoperable or at unacceptably high surgical risk, the equivocal results observed in the intermediate surgical risk group with varied study types were of notable concern. These differences in clinical evidence could confer different levels of confidence, particularly when informing funding policy decisions.

From the healthcare system perspective, both clinical and economic evidence would need to be considered when making funding policy decisions [8]. Cost-effectiveness studies may be conducted to estimate and compare the costs and effects of different interventions for treatment of aortic stenosis. Although some jurisdictions have recommended funding for TAVI use in intermediate risk patients, there is considerable uncertainty which persists from global variations in device and procedure costs, limited long-term clinical data and heterogeneous economic evidence. Analyses reported to date are predominantly based on two-year trial outcomes, and assumptions about extrapolated long-term outcomes can have substantial impacts on estimates of cost-effectiveness [15–20]. The aim of this study is to assess the cost-effectiveness of TAVI compared with SAVR in patients with intermediate surgical risk from the Singaporean healthcare system perspective using local cost inputs and clinical evidence from the most up-to-date clinical trials with published time-to-event data.

## Methods

### Patients and intervention

A three-state Markov model was developed to evaluate the cost-effectiveness of TAVI compared with SAVR in patients with severe AS of intermediate surgical risk. Clinical inputs were derived from the PARTNER 2A randomized trial [10, 11], where the intervention considered was TAVI (balloon-expandable SAPIEN XT heart-valve system, Edwards Lifesciences) and the comparator was SAVR. The most appropriate comparator was SAVR for treatment in patients at intermediate surgical risk in local clinical practice, in line with published clinical guidelines, published pivotal RCTs, and inputs from local clinician experts [10–12, 21, 22]. Standard medical therapy and rapidly-deployed aortic valve replacement were not used as comparators in the model as the former was used mostly for inoperable patients with severe AS, while the latter was rarely used in local clinical practice.

The mean age of the trial population was 81 years; 54% were male and the average Society of Thoracic Surgeons Predicted Risk of Mortality risk score was 5.8% (where a score ranging from 4 to 8% is deemed to be intermediate risk). About 77% of patients had New York Heart Association (NYHA) Class III or IV heart failure and 66% to 69% had coronary artery disease. In the model patients were assumed to enter the model at 80 years old with clinical characteristics (age, stage of heart failure according to NYHA classification and presence of coronary artery disease) similar to key TAVI trials [10, 12, 23, 24]. To ensure the applicability of trial population characteristics to the local population, local clinician experts were consulted and this assumption was considered clinically valid.

### Model structure and key specifications

**Fig. 1:**
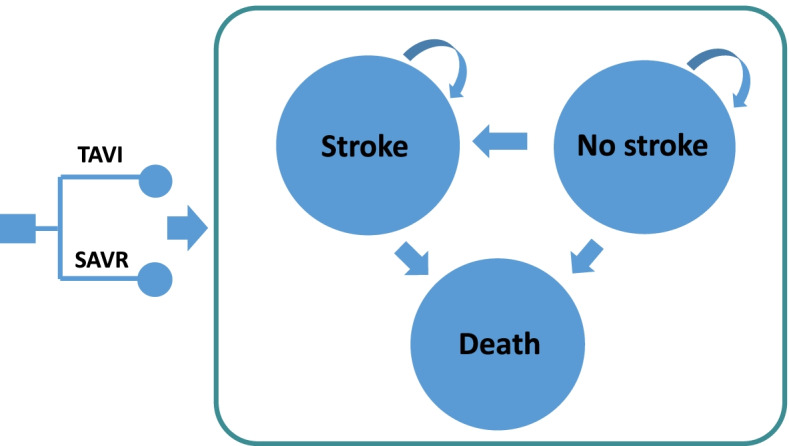
Markov state transition diagram for the economic model. Abbreviations: SAVR, surgical aortic valve replacement; TAVI, transcatheter aortic valve implantation

The microsimulation model comprised three health states: “stroke”, which represented disabling or major stroke, a serious complication with long-term sequelae; “no stroke”; and “death”, an absorbing state (Fig. 1). To ensure its face validity, it was developed in consultation with local clinical experts and referenced published economic models of TAVI which were identified via a systematic search of the literature [15–20, 25].

At the start of the simulation, patients received the index procedure of either TAVI or SAVR. A monthly cycle length was chosen to capture relevant changes in the health states. Within the first month after the procedure, patients who survived would transition to the “stroke” health state if they experienced disabling or major stroke, or transition to the “no stroke” health state if they did not. Upon transition to the “stroke” state, patients could either stay in it or move to the “death” state if they die. Patients without stroke remained in the “no stroke” state unless they experienced an episode of major stroke or died. All simulated patients were also at risk of other clinically relevant AEs – myocardial infarction (MI), major vascular complications, life-threatening or disabling major bleeding, endocarditis, new permanent pacemaker implantation, transient ischaemic attack (TIA), acute kidney injury (AKI), atrial fibrillation (AF), moderate to severe PAR, and rehospitalisation after procedure. AEs incurred per-cycle costs and disutilities.

A five-year time horizon was used in the base case to capture the costs and effectiveness outcomes, as it corresponds to the longest follow-up from PARTNER 2A, thus avoiding the need for extrapolation. This time horizon was considered appropriate given the expected life expectancy of 83.9 years in Singapore, and the approach was aligned with the Agency of Care Effectiveness Methods and Process Guide [26, 27]. A longer time horizon of 20 years was explored in a scenario analysis. Using point estimate five-year all-cause mortality data from PARTNER 2A, mortality rates between two to five years were estimated from two year published KM data assuming a linear increase over time. The effectiveness outcome used in the model is QALY. The ICERs from pairwise comparisons of incremental costs and QALYs of TAVI and SAVR were estimated. Future costs and effectiveness outcomes were discounted at 3% per annum [26]. All analyses were conducted from the Singapore healthcare system perspective, and all costs were presented in 2020 United States (US) dollars (US$1 = S$1.32) [28]. Only direct medical costs were included. Monte Carlo simulation with 10,000 iterations (seed number set at 1) was used to run the analysis using TreeAge Pro 2019, R2.1 (TreeAge Software, Williamstown, MA).

### Model inputs and model validation

Clinical outcomes that informed the base case analysis are summarised in Table 1. These included all-cause mortality, incidences of major stroke and other AEs or complications. Clinically relevant outcomes considered in the model were defined by the Valve Academic Research Consortium (VARC 2/3), consistent with endpoints reported in pivotal trials [10–12, 29, 30]. These inputs were informed by the TAVI and SAVR transfemoral-access intention-to-treat populations in PARTNER 2A trial for intermediate surgical risk patients [10, 11]. The PARTNER 2A trial was used as the base case as it was the only RCT for intermediate surgical risk group that provided the most comprehensive Kaplan–Meier (KM) curves for all-cause mortality, and AEs such as stroke (disabling and non-disabling) and moderate to severe PAR. A meta-analysis of the RCTs was not conducted as the results from different RCTs were analysed using different analytical methods. For instance, PARTNER trials used the frequentist approach for endpoint comparisons while the SURTAVI trial performed Bayesian analyses [12]. Without access to patient-level data, pooled analysis of trial data was not deemed feasible. Also, selective reporting of hazard ratios (HRs) meant that a meta-analysis would not accurately represent the collective conclusions of the trials. Where possible, the model had considered various uncertainties associated with the inputs used, and included sensitivity and scenario analyses as needed.

**Table 1:** Summary of clinical outcomes used in the base case analysis (based on PARTNER 2A trial) [10]

Clinical outcomes	TAVI (%)				SAVR (%)			
	**30 days**	**1 year**	**2 year**	**5 year**	**30 days**	**1 year**	**2 year**	**5 year**
All-cause mortality	3	10	14.2	42.7	4.1	12.3	17.2	40.5
Disabling or major stroke	2.3	4.3	5.3	8.7	4.2	6	6.7	8.3
Rehospitalisation	5.5	13.1	18.4	32	6.5	14.8	17.1	24.1
MI	0.6	1.9	3	9.4	1.8	3.2	4.2	8.1
Major vascular complication	8.5	8.8	9	9.6	3.9	4.3	4.5	5.1
Life-threatening or disabling bleeding	6.7	11.1	13.6	21.1	41.4	43.4	44.7	48.6
Endocarditis	0	0.8	1.5	3.7	0	0.9	0.9	1.9
AKI	0.5	2.2	2.5	3.4	3	5.2	6.4	10
New PPI	8.1	9.6	11.4	15.1	7.1	9.5	10.8	13.5
TIA	0.9	2.6	3.8	5.8	0.3	1.8	2.3	4.3
Atrial fibrillation	4.9	5.9	7.4	11.7	26.7	27.6	27.8	30.7
Paravalvular aortic regurgitation	3.75	3.75	8.27	6.44	0.49	0.49	0.57	0.34

*Abbreviations: AKI *acute kidney injury, *MI *myocardial infarction, *PPI *permanent pacemaker implantation, *SAVR *surgical aortic valve replacement, *TAVI *transcatheter aortic valve implantation, *TIA *transient ischaemic attack

Notes: 1. All-cause mortality and disabling or major stroke were extracted from Kaplan–Meier curves presented in Leon et al. (2016) for intermediate-risk surgical patients [10]

2. Clinical outcomes data were available for only two years in transfemoral population for all variables except all-cause mortality, disabling or major stroke, repeat hospitalisation, TIA and paravalvular aortic regurgitation; beyond two years, the increased rates in incidence were assumed to be based on the last observed data for acute kidney injury, major bleeding and major vascular complication, or the same as that reported in pooled population (transfemoral and non-transfemoral) between 2^nd^ and 5^th^ year for atrial fibrillation, endocarditis, myocardial infarction and pacemaker implantation

3. Let *RR*_*S*_ be the relative risk for death due to having a stroke, *M*_*NS*_ and *M* be the mortality rate for those without stroke and the entire cohort respectively, whilst *X* is the proportion of the cohort with stroke. The adjusted mortality values are: *M* = X*RR_S_*M_*NS*_ + (1 - *X*)*M_*NS*_. This may be re-arranged to give *M*_*NS*_ = *M*/[X*RR_*S*_ + (1 - *X*)], as the mortality for those without stroke is the unknown

Transitions between health states were determined by all-cause mortality and the incidence of major stroke. All probabilities of clinical outcomes were based on KM estimates at the specific time points. Where clinical outcomes (e.g. all-cause mortality and major stroke) were presented as KM curves, survival data were extracted using WebPlotDigitizer, a web-based digitizer programme [31]. For KM probabilities that were only available at specific time points (e.g. 30 days, 1 year, 2 years, 3 years and 5 years after the index procedure) in the studies, the monthly incidence rate of clinical events was assumed to be constant between each time point. The monthly transition probability of each clinical outcome was calculated using the formula 1- S(t-1)/S(t), where S(t) was the survival probability of the event at time t.

Patients with stroke were assumed to be at a higher risk of death relative to those without stroke. Due to the lack of data in patients with intermediate surgical risk, the elevated all-cause mortality risk for patients in the “stroke” health state was derived from PARTNER 1B and 1A analyses [32] on patients who were inoperable or had high surgical risk respectively. To obtain lower mortality rates for the “no stroke” health state, all-cause mortality was divided by the weighted HRs of death of patients with and without stroke at each time point (see Table 2).

**Table 2:** Hazard ratios of all-cause mortality in patients with versus without stroke (cycle 1 to 15)

Cycle	1	2	3	4	5	6	7	8	9	10	11	12	13	14	15
HR	8.5	4.72	3.58	2.97	2.63	2.21	2.15	1.83	1.86	1.5	1.51	1.42	1.22	1.19	1.16

*Abbreviation: HR* hazard ratio

Notes: 1. The incidence, timing, risk factors and outcomes of neurological events (stroke and TIA) after TAVI were analysed in the high-risk and inoperable cohorts of the PARTNER trial. Time-related events, including mortality following a neurological event, were then estimated by the Kaplan–Meier method (from Kapadia et al., 2016) [32]. HRs of mortality in patients with and without stroke were then derived using data on survival after stroke and expected survival had a stroke not occurred

The risks of other AEs were assumed to be the same in the “stroke” and “no stroke” health states. HRs for mortality in the “stroke” health state were assumed to be the same regardless of the type of procedure received. Following a stroke event, patients who enter the “stroke” health state were at an increased risk of death compared with their age-specific counterparts who have not experienced a stroke up until 15 months; beyond 15 months, it was assumed that there was no difference in the hazard of death between patients in the “stroke” and “no stroke” health states and patients in the “stroke” health state would revert to background population mortality.. This assumption was corroborated with local clinician experts, other published CEAs, and the Markov traces used in model validation (see Additional materials file 1) [19, 20, 33].

The impact of AEs on mortality was assumed to have been accounted for in all-cause mortality. Markov traces of all-cause mortality generated for model validation showed that the simulated KM curves were very similar to that observed in the PARTNER 2A trial (see Additional materials file 1).

### Resources and costs

Consistent with the healthcare system perspective, only direct medical costs were considered in the model. These included charges of index procedures of TAVI or SAVR, follow-up visits and treatment for AEs. After the index hospitalisation, patients incurred costs for outpatient follow-up care, which was set at one cardiologist visit per year. It was assumed that patients who experienced AEs would incur a one-time treatment cost. Charges of a follow-up visit every three months were only assigned in the “stroke” health state.

Table 3 showed the charges applied in the model extracted using relevant Australian Classification of Health Interventions (ACHI) codes and International Classification of Diseases (ICD)-10 codes (details in Additional materials file 2). The aggregated mean charges of respective index procedures and treatment of AEs were sourced from inpatient episodes admitted between 2016 and 2018 from the Singapore Ministry of Health (MOH) Casemix and Subvention System, using their respective ACHI codes for TAVI, SAVR, and permanent pacemaker implantation, and ICD-10 codes for other AEs. Codes were determined based on World Health Organization ICD-10 online version and published literature, which were then validated by a clinical coder. Charges of outpatient follow-up care after index procedure were based on estimates from the survey of local experts. The charge of a neurologist outpatient visit was obtained from MOH subvention data. Charges for outpatient follow-up applied in the model were converted to monthly costs for the analysis. As all costs used were assumed to be in 2020 Singapore dollars and converted to 2020 US dollars using the 2020 exchange rate [28], inflation was not considered.

**Table 3:** Cost parameters and corresponding values

Parameter	Cost per episode1, mean ± SD (95% CI) 2020 US$
TAVI mean episodic charge without AEs	54,301
SAVR mean episodic charge without AEs	26,109
Follow-up care after procedure	372
Disabling or major stroke	14,243 ± 11,914 (4252 to 47,508)
Neurologist outpatient visit	243
Rehospitalisation	6120 ± 9280 (667 to 26,476)
MI	13,736 ± 13,143 (1436 to 44,309)
Major vascular complication	13,773 ± 13,858 (3486 to 54,403)
Life-threatening, disabling, or major bleeding	8652 ± 7524 (2408 to 30,786)
Endocarditis	26,952 ± 29,945 (1813 to 114,305)
AKI	6203 ± 8445 (711 to 28,414)
New PPI	13,384 ± 8805 (5856 to 34,942)
TIA	2842 ± 2213 (749 to 7819)
Atrial fibrillation	5152 ± 8535 (597 to 25,330)
Paravalvular aortic regurgitation	22,231 ± 20,436 (831 to 69,572)

*Abbreviations: AEs* adverse events; *AKI* acute kidney injury, *CI* confidence interval, *MI* myocardial infarction, *PPI* permanent pacemaker implantation, *SAVR* surgical aortic valve replacement, *SD* standard deviation, *TAVI* transcatheter aortic valve implantation

Note: 1. The above costs were retrieved from the Casemix and Subvention System, which contains inpatient, day surgery episodic data or specialist outpatient visit data from public healthcare institutions (PHIs) for subvention purpose. It contains information on patient demographics, episode or visit details including ward class (admitted/discharged), subsidy status (i.e. subsidised/private), length of stay, cost (i.e. total charge, total bill), and clinical data such as diagnosis and procedure codes

2. More details on Australian Classification of Health Interventions (ACHI) and International Classification of Diseases (ICD)-10 codes and methods used are in Additional materials file 2

### Health state utilities

Table 4 showed the EQ-5D utilities from the PARTNER 2A trial used in the base case [34]. In the model, patients started with the first-month utility in the first cycle. To capture the gradual change in patient’s quality of life, utility was assumed to change linearly over time. For example, the change from Month-1 utility to the Month-12 utility occurred in equal increments or decrements. As the trial did not report utility data beyond 24 months, it was assumed that there was no difference in utilities between TAVI and SAVR patients beyond 24 months, and the last utility value applied in the TAVI group was assigned to both groups in subsequent cycles. As the impact of AEs on quality of life was assumed to be already incorporated in the utility values reported in the trials, no additional utility decrements associated with AEs were applied in the base case analysis to avoid double-counting.

**Table 4:** Utility values and utility decrements

A.EQ-5D utility values applied in base case			
Time point	TAVI, mean ± SD (95% CI)	SAVR, mean ± SD (95% CI)	Source
Baseline	0.75 ± 0.17	0.73 ± 0.17	[34], utility weights were obtained from patients eligible for transfemoral approach
Month-1	0.81 (0.79 to 0.82)	0.73 (0.71 to 0.74)	
Month-12	0.79 (0.78 to 0.81)	0.80 (0.78 to 0.81)	
Month-24	0.78 (0.76 to 0.79)	0.77 (0.75 to 0.79)	

*Abbreviations: CI *confidence interval, *SAVR *surgical aortic valve replacement, *SD *standard deviation, *TAVI *transcatheter aortic valve implantation

### Sensitivity analyses

One-way sensitivity analysis (OWSA) was conducted over the range of predefined values for specific model parameters (i.e. 95% confidence interval or ± 20% for all variables except discount rates which varied from 0 to 5%). The parameters included in the OWSAs were discount rate, implant costs, costs of procedure episode, costs for treatment of AEs and health state utilities. The impact on the ICER was presented in a Tornado diagram.

A multivariate probabilistic sensitivity analysis was performed, using 5,000 s-order Monte Carlo simulations (10,000 first-order simulation trials). Uncertainty in model inputs were explored by randomly sampling the parameters from assigned distributions (see details in Additional materials file 3). All-cause mortality and incidence of AEs in the first month and utility values were modelled as beta distribution, whereas costs of AE treatments were assumed to follow gamma distributions. A cost-effectiveness acceptability curve was generated to present the probability of each strategy being cost-effective at willingness-to-pay (WTP) thresholds.

### Scenario analyses

To test the robustness of the model results to data and methodological assumptions, four scenario analyses were examined – Scenario 1: Clinical outcomes using SAPIEN 3 valve (PARTNER S3i PSM); Scenario 2: Clinical outcomes using CoreValve® System (SURTAVI), Scenario 3: Lifetime horizon of 20 years applied on base case setting; Scenario 4: Incorporating disutilities associated with AEs in base case. More details on the scenarios are in Additional materials file 4.

## Results

### Base case analysis

Table 5 showed that the base case ICER for TAVI at intermediate surgical risk was US$315,760 per QALY gained. This was based on average total discounted costs incurred per patient of US$70,959 for TAVI and US$39,492 for SAVR, and patients receiving TAVI experienced 0.10 more discounted QALYs than those receiving SAVR.

**Table 5:** Base case and probabilistic sensitivity analysis results

Comparison	Costs, 2020 US$	Incremental costs	QALYs	Incremental QALYs	ICER (cost per QALY), 2020 US$
A.Base case					
TAVI	70,959	31,467	2.92	0.10	315,760
SAVR	39,492	-	2.82		
A.Probabilistic sensitivity analysis					
TAVI	70,997	31,541	2.92	0.10	319,241
SAVR	39,456	-	2.82	-	-

Abbreviations: *ICER* Incremental cost-effectiveness ratio, *QALY* Quality-adjusted life year, *SAVR* Surgical aortic valve replacement, *TAVI* Transcatheter aortic valve implantation

### Sensitivity analyses

**Fig. 2:**
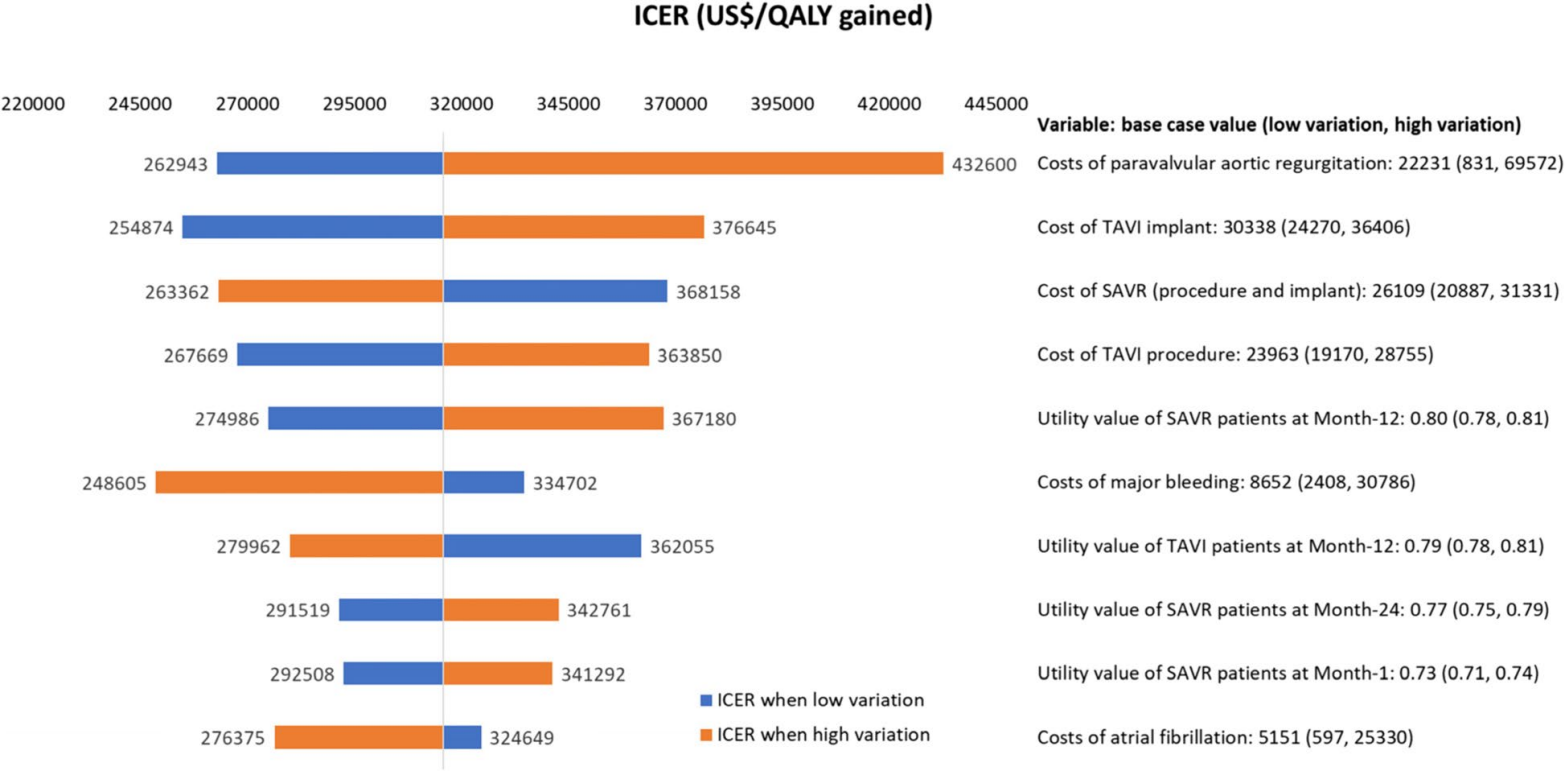
Tornado diagram for deterministic one-way sensitivity analysis (top 10 drivers)

Results from deterministic OWSA showed that the ICERs were most sensitive to the cost of PAR, cost of TAVI implant, cost of SAVR procedure and implant, cost of TAVI procedure, and the utility value of SAVR patients at Month-12 (see Fig. 2).

**Fig. 3:**
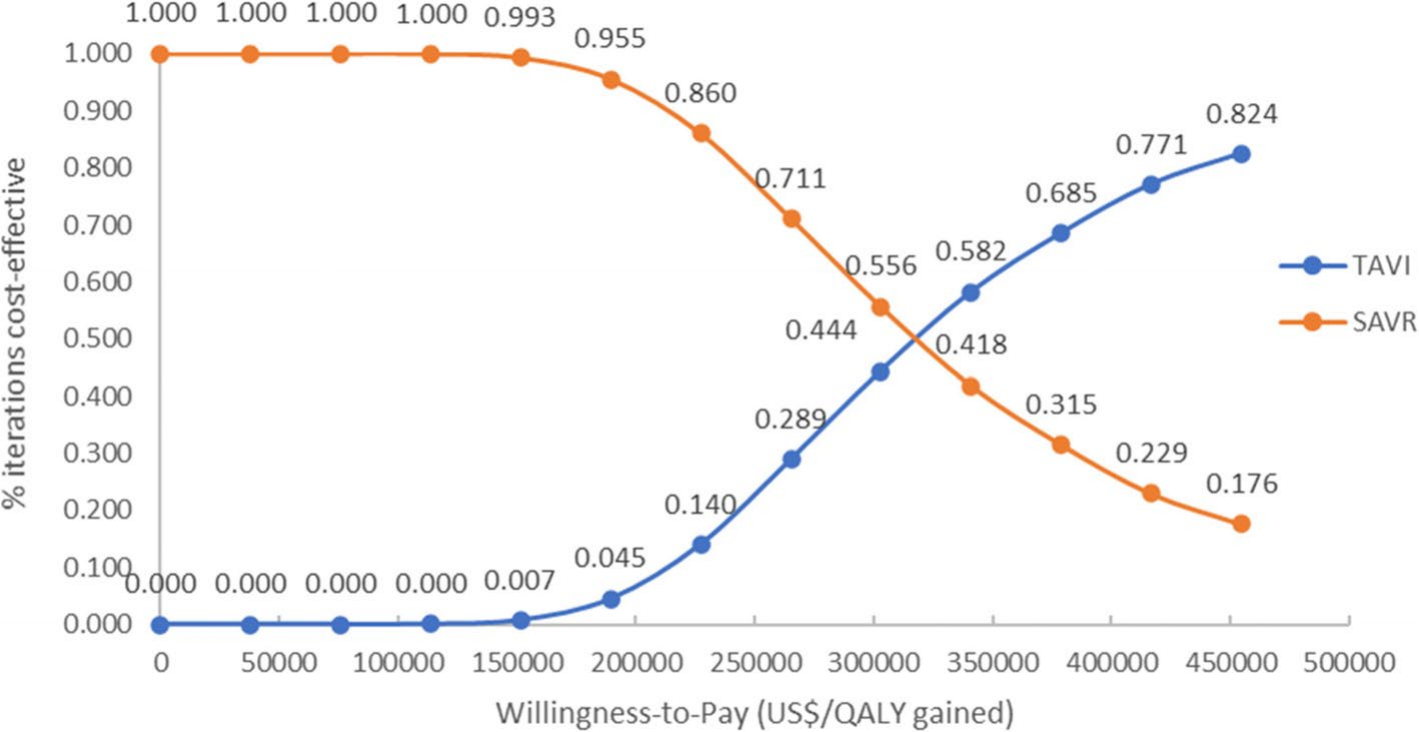
Cost effectiveness acceptability curve at varying willingness-to-pay levels for TAVI versus SAVR in intermediate surgical risk patients

The probabilistic sensitivity analysis results showed that the mean probabilistic ICER was US$319,241 per QALY gained, which was similar to US$315,760 per QALY gained in the deterministic base case results (see Table 5). Only when the threshold exceeded approximately US$321,970 (S$425,000) per QALY gained did TAVI become the more cost-effective option (see Fig. 3).

### Scenario analyses

The ICER reduced significantly to US$86,337 per QALY gained in Scenario 1, where clinical outcomes for the SAPIEN 3 valve in the PARTNER S3i PSM study were used, due to the smaller incremental costs and greater incremental QALYs gained. In Scenario 2 when clinical outcomes were based on SURTAVI trial which used TAVI CoreValve® System, a much higher ICER of US$837,595 per QALY gained was estimated as the QALY gain was only 0.04. When the time horizon was increased from 5 to 20 years in Scenario 3, the TAVI intervention was dominated by SAVR as QALYs associated with SAVR was higher than TAVI at 0.31. When disutilities associated with AEs were incorporated, TAVI became slightly more favourable with a 5% reduction in ICER, at US$300,070 per QALY gained. Collectively, these scenario analyses suggested the ICER was highly sensitive to both the source of effectiveness data and the time horizon, but not to AE-related disutilities. More details are in Additional materials file 4.

## Discussion

To the best of our knowledge, this is the first cost-effectiveness analysis that comprehensively considered the most up-to-date published trial data with time-to-event data including the PARTNER 2A, PARTNER S3i PSM, and SURTAVI trials. The use of scenario analyses also allowed an assessment of the impact on ICERs when the clinical trials, assumptions, and time horizons were varied.

With a base case ICER estimated to be US$315,760 per QALY gained, the use of TAVI in patients with intermediate surgical risk would unlikely be deemed cost-effective by conventional standards. Based on recognised reference international cost-effectiveness thresholds such as from the National Institute for Health and Clinical Excellence (NICE) and the World Health Organisation (WHO), the base case ICER obtained suggests that TAVI is unlikely to be cost-effective in this patient group [35–39]. The high ICER was attributed to the high implantation and procedure costs for TAVI relative to SAVR, and a marginal cumulative improvement of 0.10 QALYs as the simulated mortality in TAVI exceeded SAVR at around 45 months (3.75 years) post-implantation. When uncertainty was considered, deterministic one-way sensitivity analysis showed that ICERs continued to be high, ranging from US$248,605 to US$432,600 per QALY gained. Although there is no known ICER threshold in Singapore, ICERs for medical technologies previously recommended for subsidy in Singapore were generally below US$34,091 (S$45,000) per QALY gained [26, 40]. When US$34,091 was used as WTP in the model, an incremental net monetary benefit (INB) analysis culminated in a negative INB (-US$28,097; lower limit -US$11,133, higher limit -US$45,061), indicating that TAVI was unlikely a cost-effective option in Singapore when compared with SAVR [40]. This was also consistent with the probabilistic sensitivity analyses where TAVI became the more cost-effective option only when WTP exceeded approximately US$321,970 (S$425,000) per QALY gained.

The scenario analyses showed significant changes in ICER when the source of clinical trial data or time horizon were varied. The one-year outcomes data on PARTNER S3i PSM were used in Scenario 1 of this study instead of a conference abstract on its five-year outcomes data as no KM curves on all-cause mortality and stroke were available in the latter [14]. Nevertheless, the ICER estimated using observed five-year data from the PARTNER S3i PSM is likely to be higher than that estimated using the one-year data. This can be inferred from the higher reported all-cause mortality in TAVI at five years of 39.1% compared to 26.8% extrapolated from one-year data from the PARTNER S3i PSM, and relatively similar reported and extrapolated all-cause mortality at five years for SAVR (40.8% vs 41.3% respectively). PARTNER S3i PSM data showed that early all-cause mortality benefits from TAVI over SAVR at one year (TAVI 6.5% vs SAVR 12.2%) diminished at five years (TAVI 39.1% vs SAVR 40.8%), depicting how simulated data could differ from actual data as the expected trajectory of effects changes during the simulated period and early benefits did not persist in the reported trial results. It was difficult to determine if this discrepancy could also be influenced by the lack of randomisation in PARTNER S3i PSM. Similarly, for the CoreValve® System in SURTAVI trial, given the lack of utility data and clinical evidence beyond 24 months, the use of PARTNER 2A utility weights and the assumption that patients in both arms would follow linearly projected risks based on the second-year probability in the SAVR arm may have attributed to the higher ICER. We observed that the simulated mortality benefit conferred by CoreValve® System compared with SAVR was less than that in our base case, as shown in Additional materials file 1, where the probability of death in TAVI arm overtook that of SAVR arm from Month-11, until Month-24.

When compared with another published cost-effectiveness analysis (CEA) study on TAVI in intermediate risk study in Singapore, results from our study were much higher than their reported ICER of US$25,631 per QALY gained over a five-year time horizon despite also using five-year follow-up PARTNER 2A trial data [9]. These differences could be attributed to differences in model structure, health utilities, and cost differential between TAVI and SAVR. Unlike the published 2020 Singapore study, our study used only stroke health states and cost parameters for other AEs as published RCT data could not support mutually exclusive health states for different AEs [9]. While our model used a higher cost differential between TAVI and SAVR based on extractions from the national database than the 2020 study [9], the incremental QALY gain difference in our model was only about half of that from the 2020 model (0.10 QALYs vs 0.19 QALYs). For disutilities of AEs, the published 2020 study assumed a long-term disutility of 0.24 for stroke which was larger than the disutility value 0.161 used in the scenario analyses in our study, and ongoing AKI disutility was applied as a one-off occurrence in our study[9]. Furthermore, the scenario analyses in our study suggested that TAVI was worse off than SAVR based on the extrapolated trajectory for all-cause mortality beyond five years from the PARTNER 2A trial and became dominated when time horizon increased from five to 20 years. However, the published 2020 study reported lower ICER when time horizon increased from five to eight years [9]. Given the extrapolated trajectory, based on our model, it is unlikely that the ICER would be more favourable for TAVI when the time horizon increases. More details are in Additional materials file 5.

Beyond Singapore, CEA studies on TAVI in intermediate surgical risk patients in Australia [33], Canada [15, 17, 20], France [16], Japan [25], Ireland [41], Italy [42], Norway [43], Scotland [44], Spain [45], the United States (US) [8] and Wales [46] have been published. Published ICERs varied, with some reporting dominant ICERs favouring TAVI [8] while other studies reported ICERs as high as US$134,775 (£98,965) per QALY gained [44], showing inconsistency in cost-effectiveness of TAVI in the intermediate surgical risk group. Our study and the published 2020 study from Singapore used five year all-cause mortality data from PARTNER 2A and mortality rates between two to five years were estimated by assuming a linear increase over time [9]. All other published CEA studies had used one- to two-year data for TAVI from PARTNER 2A, PARTNER S3i registry, SURTAVI or OCEAN-TAVI registry, or a combination of them[8, 16, 17, 25, 33, 41–44, 46]. Extrapolation techniques for clinical inputs post two-year follow-up varied among the studies, with different studies making different assumptions on risks of complications and mortality. Studies that extrapolated all-cause mortality from the two-year PARTNER 2A trial yielded ICERs that were more favourable to TAVI as the extrapolated trajectory would extend TAVI’s early benefits on all-cause mortality. Similar to our study, costs relating to device, procedure, or both of TAVI and SAVR were consistently identified as key driver in the studies [15, 20, 43]. Data used to generate ICERs in these studies were limited to PARTNER 2A and did not reflect clinical outcomes of the latest generation TAVI device. A larger cost differential between SAVR and the more expensive TAVI would yield higher ICERs. It is plausible that the cost-effectiveness of TAVI in intermediate risk patients may change should the cost of TAVI be reduced

Globally, various health technology assessment agencies have evaluated TAVI in patient with intermediate surgical risk [7, 41, 43, 44, 46–49]. Health technology assessment agencies that performed de novo CEA – Healthcare Improvement Scotland (HIS), Health Technology Wales (HTW), Health Information and Quality Authority (HIQA) of Ireland, Norwegian Institute of Public Health (NIPH) – yielded ICERs that ranged from dominant ICERs favouring TAVI [41]to ICERs ranging from 1.04 million Krones to £98,965 (US$119,097 to US$134,775)[43, 44, 46]. All of them had used two-year follow-up data from PARTNER 2A which would have overestimated TAVI’s benefit as the early all-cause mortality benefits of TAVI were assumed to persist in the extrapolated trajectory for the simulated time horizon from two years to lifetime. When applied over longer time horizons of 15 years to lifetime, the ICERs would favour TAVI to an even greater degree, albeit with greater uncertainty given the lack of robust comparative evidence of the latest generation TAVI device for consideration in these models. Findings from these health technology assessment agencies had led to positive reimbursement decisions in three of the four jurisdictions. Had more mature five-year PARTNER 2A been available and incorporated in their assessments, higher ICER ranges would be expected. This exemplifies a key challenge faced by health technology assessment agencies when the evidence continues to evolve in a different trajectory or direction after reimbursement decisions are made and how often reassessment should take place when new or long-term evidence emerges.

From the payer perspective in Singapore, successful implementation of a positive reimbursement decision for TAVI for specific surgical risk groups is contingent on objective surgical risk assessment and susceptibility to leakage into lower risk groups. Locally, the likelihood of unintended leakage into lower surgical risk groups could yield poorer cost-effectiveness results due to the narrowing effectiveness between the intervention and comparator over time, and the need for valve replacement when implanted in patients in lower risk groups who tend to be younger. Although STS risk scores could be used to objectively determine the level of surgical risk, other clinical and patient factors such as frailty could also affect the multidisciplinary heart team’s determination of surgical risks assessment.

There were several limitations in this study. First, the published clinical evidence from PARTNER 2A and SURTAVI may not sufficiently reflect the current generation of TAVI models, current TAVI operator experience, and more rigorous patient selection for TAVI over time. Although PARTNER 2A and SURTAVI provided the most robust five- and two-year comparative evidence, they mainly used earlier generation TAVI models that were no longer used in current local clinical practice. Except for greater early quality of life improvement at one month which diminished by one year from current generation SAPIEN 3 model when compared to predicate SAPIEN XT from PARTNER S3i PSM [50], there was limited head-to-head comparative evidence in intermediate surgical risk patients to show different clinical outcomes between current and older generation TAVI. Second, although PARTNER S3i PSM used SAPIEN 3, a device that is currently offered in local clinical practice, it was difficult to determine if the risk of residual bias in the PSM was fully eliminated as there was insufficient published information on whether the final models included relevant interactions and higher order terms, and whether the distribution of baseline covariates was balanced within each propensity score quintile. Third, due to a lack of all-cause mortality data that differentiated patients who developed stroke from those who did not, hazard ratios from other PARTNER trials on high surgical risk or inoperable patients had to be applied to meet this data need [32]. However, these patients had inherently different level of surgical risk as our modelled population and the actual hazard ratios might differ. Although hazard ratio estimates based on actual survival curves from patients with intermediate surgical risk that did not develop stroke could improve the validity of these estimates, the availability of granular patient-level data would be needed to validate the assumption that the risk of adverse events would be the same for patients with and without stroke. Fourth, utility values for the “stroke” and “no stroke” health states were assumed to be the same due to the lack of specific utility estimates for patients with and without disabling or major stroke in the AS population. In the absence of longer-term utility data, the utility weights beyond follow-up were assigned based on the last observed value. However, we observed in the Markov traces that the largest difference in proportion with stroke between TAVI and SAVR was at most 2% at any point during the time horizon, while the proportion without stroke was almost the same. Hence, any potential difference in utility values between “no stroke” and “stroke” state would not lead to significant difference in incremental QALYs as shown in Scenario 4.

## Conclusion

From the Singapore healthcare system perspective, the inclusion of longer-term trial data and the large cost difference between TAVI and SAVR ascertained that in patients with intermediate surgical risk, TAVI is unlikely to be cost-effective by conventional standards. Evolving and long-term evidence has the potential to significantly impact CEA findings and outcomes of reimbursement decisions.

## Supplementary Information

**Additional file 1: Figure S1-1.** Actual death proportion from PARTNER 2A trial and simulated Markov traces in the economic model. **Figure S1-2.** Simulated Markov traces for TAVI and SAVR arms in SURTAVI trial for intermediate surgical risk.

**Additional file 2: Table S2-1.** Cost parameters and corresponding codes

**Additional file 3: Table S3-1.** Parameters, distributions and upper and lower values used in probabilistic sensitivity analyses

**Additional file 4:Table S4-1.** Summary of clinical outcomes used in Scenario 1 (based on PARTNER S3i PSM). **Table S4-2.** Utility values and decrements applied in scenario analyses. **Table S4-3.** Summary of clinical outcomes used in Scenario 2 (based on SURTAVI trial). **Table S4-4.** Results of scenario analyses.

**Additional file 5.** Comparison of current model with local published 2020 study [9].

## Data Availability

The dataset(s) supporting the conclusions of this article are included within the article and (and its additional file(s)).

## Abbreviations

ACHI: Australian classification of health interventions
AE: Adverse Events
AF: Atrial fibrillation
AKI: Acute kidney injury
AS: Aortic Stenosis
AVR: Aortic valve replacement
EQ-5D: EuroQol-5 Dimension
HR: Hazard ratio
ICD: International classification of diseases
ICER: Incremental cost-effectiveness ratio
KM: Kaplan-Meier
MI: Myocardial infarction
MOH: Ministry of Health Singapore
NICE: The National Institute for Health and Care Excellence
NYHA: New York Heart Association
PAR: Paravalvular aortic regurgitation
PSM: Propensity-scored matched studies
QALY: Quality-adjusted life year
RCT: Randomised controlled trial
SAVR: Surgical aortic valve replacement
TAVI: Transcatheter aortic valve implantation
TIA: Transient ischaemic attack
US: United States
WHO: World Health Organisation
WTP: Willingness-to-pay
