# Supplementary material for: Cost-effectiveness of transcatheter aortic valve implantation in patients with severe symptomatic aortic stenosis of intermediate surgical risk in Singapore
Source: BMC Health Serv Res. 2022 Aug 4;22:994. doi: 10.1186/s12913-022-08369-5 (PMC9354430; doi:10.1186/s12913-022-08369-5)
Supplement: Supplementary file 1 — Additional file 1: Figure S1-1. Actual death proportion from PARTNER 2A trial and simulated Markov traces in the economic model. Figure S1-2. Simulated Markov traces for TAVI and SAVR arms in SURTAVI trial for intermediate surgical risk. [file 12913_2022_8369_MOESM1_ESM.docx]

# ADDITIONAL MATERIALS

**Additional materials file 1**

**Figure S1-1. Actual death proportion from PARTNER 2A trial and simulated Markov traces in the economic model**

**
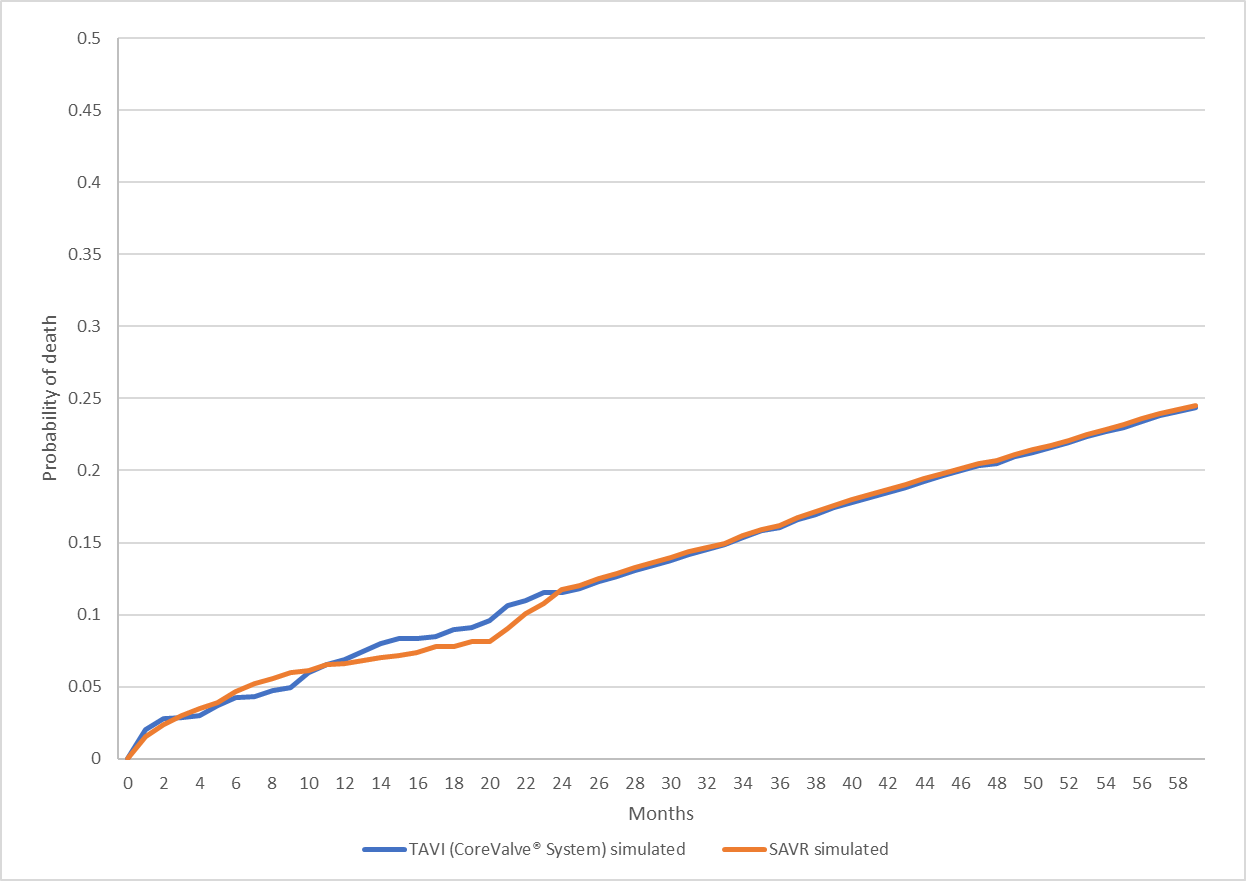
**

Figure S1-2. Simulated Markov traces for TAVI and SAVR arms in SURTAVI trial for intermediate surgical risk
