# Supplementary material for: Cost-effectiveness of transcatheter aortic valve implantation in patients with severe symptomatic aortic stenosis of intermediate surgical risk in Singapore
Source: BMC Health Serv Res. 2022 Aug 4;22:994. doi: 10.1186/s12913-022-08369-5 (PMC9354430; doi:10.1186/s12913-022-08369-5)
Supplement: Supplementary file 2 — Additional file 2: Table S2-1. Cost parameters and corresponding codes [file 12913_2022_8369_MOESM2_ESM.docx]

Additional materials file 2

Table S2-1. Cost parameters and corresponding codes

| **Parameter** | **Codes** |
| --- | --- |
| TAVI mean episodic charge without AEs | ACHI code: 3848000, 3848100, 3848800, 3848900, 3848901 |
| SAVR mean episodic charge without AEs | ACHI code: 3827001 |
| Follow-up care after procedure | - |
| Disabling or major stroke | ICD-10: I629, I63, I630, I631, I632, I633, I634, I635, I636, I638, I639 |
| Neurologist outpatient visit | - |
| Rehospitalisation | ICD-10: I200, I209, I500, I501, I509 |
| MI | ICD-10: I21, I210, I211, I212, I213, I214, I219 |
| Major vascular complication | ICD-10: A419, I358, I458, I509, I7020, I719, I723, I724, I742, I743, I744, I745, I770, I771, I802, I99 |
| Life-threatening, disabling, or major bleeding | ICD-10: K922 |
| Endocarditis | ICD-10: I33, I330, I339, I38 |
| AKI | ICD-10: N17, N170, N171, N172, N178, N179, N19 |
| New PPI | ACHI code: 3835300, 3835000 |
| TIA | ICD-10: G45, G450, G451, G452, G458, G459 |
| Atrial fibrillation | ICD-10: I48, I480, I481, I489, |
| Paravalvular aortic regurgitation | ICD-10: I351 |

Abbreviations: ACHI, Australian Classification of Health Interventions; AE, adverse event; AKI, acute kidney injury; ICD, International Classification of Diseases; MI, myocardial infarction; PPI, permanent pacemaker implantation; SAVR, surgical aortic valve replacement; TAVI, transcatheter aortic valve implantation; TIA, transient ischemic attack

Notes:

1. Charges in the initial index procedure were associated with implant, surgery facility, surgeon, surgery, anaesthetist and hospitalisation in regular ward and ICU. Costs for outpatient follow-up care comprised expenditures of electrocardiogram assessment and clinician consultation.
2. The Casemix and Subvention System contains inpatient, day surgery episodic data or specialist outpatient visit data from public healthcare institutions (PHIs) for subvention purpose. It contains information on patient demographics, episode or visit details including ward class (admitted/discharged), subsidy status (i.e. subsidised/private), length of stay, cost (i.e. total charge, total bill), and clinical data such as diagnosis and procedure codes.
3. Only cases without any adverse events were applied in the model for the cost estimation of index procedure.
4. Treatment costs of adverse events were identified using corresponding codes as the primary code. For episodes of disabling or major stroke, major vascular complication, and life-threatening, disabling, or major bleeding to be included in the analyses, length of stay had to be more than seven days, seven days, and five days respectively. These filters were based on the clinicians estimates of average length of stay for the various adverse events.
5. Case definition for TAVI: Any inpatient episode with the following ACHI codes admitted between 2016 and 2018 in patients who are Singapore citizens or permanent residents (SC/PR) aged ≥75 to <90 years with an implant charge ≥S$20K to ≤S$50K, excluding those who are transferred for admission, discharged against advice and social overstayers. Additional filters for costs including AEs included those with adverse events of interest but excluding cases with total charge >S$90K (TAVI); additional filters for costs without AEs excluded those with adverse events of interest and total charge >S$90K (TAVI).
6. Case definition for SAVR: Any inpatient episode with the following ACHI codes admitted between 2016 and 2018 in patients who are SC/PR aged ≥60 years with an implant charge ≥S$1K to ≤S$10K, excluding those who are transferred for admission, discharged against advice and social overstayers. Additional filters for costs including AEs included those with adverse events of interest but excluding cases with total charge >S$50K (SAVR); additional filters for costs without AEs excluded those with adverse events of interest and total charge >S$50K (SAVR).
