# Supplementary material for: Cost-effectiveness of transcatheter aortic valve implantation in patients with severe symptomatic aortic stenosis of intermediate surgical risk in Singapore
Source: BMC Health Serv Res. 2022 Aug 4;22:994. doi: 10.1186/s12913-022-08369-5 (PMC9354430; doi:10.1186/s12913-022-08369-5)
Supplement: Supplementary file 3 — Additional file 3: Table S3-1. Parameters, distributions and upper and lower values used in probabilistic sensitivity analyses [file 12913_2022_8369_MOESM3_ESM.docx]

**Additional materials file 3**

**Table S3-1. Parameters, distributions and upper and lower values used in probabilistic sensitivity analyses**

| Model parameter | Probability distribution | Distribution parameters | Mean/base case | Lower limit | Upper limit | |
| --- | --- | --- | --- | --- | --- | --- |
| **Costs** | | | | | | |
| TAVI implant | Gamma | - | US$30338 | ±20% | | |
| TAVI procedure | Gamma | - | US$23963 | ±20% | | |
| SAVR (implant and procedure) | Gamma | - | US$26109 | ±20% | | |
| Disabling or major stroke | Gamma | $\alpha=1.43, \beta=7.6$ | US$14243 | US$4252 | | US$47508 |
| Rehospitalisation | Gamma | $\alpha=0.43, \beta=5.38$ | US$6120 | US$666 | | US$26476 |
| MI | Gamma | $\alpha=1.09, \beta=6.02$ | US$13736 | US$1436 | | US$44309 |
| Major vascular complication | Gamma | $\alpha=0.99, \beta=5.43$ | US$13773 | US$3486 | | US$54403 |
| Life-threatening, disabling, or major bleeding | Gamma | $\alpha=1.32$, $\beta=1.16$ | US$8652 | US$2408 | | US$30786 |
| Endocarditis | Gamma | $\alpha=0.81$, $\beta=2.28$ | US$26952 | US$1813 | | US$114305 |
| AKI | Gamma | $\alpha=0.54$, $\beta=6.59$ | US$6203 | US$711 | | US$28414 |
| New PPI | Gamma | $\alpha=2.31$, $\beta=1.31$ | US$13384 | US$5856 | | US$34942 |
| TIA | Gamma | $\alpha=1.65, \beta=4.4$ | US$2842 | US$749 | | US$7819 |
| Atrial fibrillation | Gamma | $\alpha=0.36, \beta=5.36$ | US$5152 | US$597 | | US$25330 |
| Paravalvular aortic regurgitation | Gamma | $\alpha=1.18, \beta=4.03$ | US$22231 | US$831 | | US$69572 |
| **30-day transition probability in TAVI** | | | | | | |
| All-cause mortality | Beta | $\alpha=22.22,$ $\beta=750.78$ | 0.03 | - | | |
| Disabling or major stroke | Beta | $\alpha=17.8,$ $\beta=756.2$ | 0.023 | - | | |
| Rehospitalisation | Beta | $\alpha=42.57,$ $\beta=731.43$ | 0.055 | - | | |
| MI | Beta | $\alpha=4.64,$ $\beta=769.36$ | 0.006 | - | | |
| Major vascular complication | Beta | $\alpha=65.79,$ $\beta=708.21$ | 0.085 | - | | |
| Life-threatening, disabling or major bleeding | Beta | $\alpha=51.86,$ $\beta=722.14$ | 0.067 | - | | |
| AKI | Beta | $\alpha=3.87,$ $\beta=770.13$ | 0.005 | - | | |
| New PPI | Beta | $\alpha=62.69,$ $\beta=711.31$ | 0.081 | - | | |
| TIA | Beta | $\alpha=6.97,$ $\beta=767.03$ | 0.009 | - | | |
| Atrial fibrillation | Beta | $\alpha=37.93,$ $\beta=736.07$ | 0.049 | - | | |
| Paravalvular aortic regurgitation | Beta | $\alpha=32.69,$ $\beta=838.31$ | 0.0375 | - | | |
| **30-day transition probability in SAVR** | | | | | | |
| All-cause mortality | Beta | $\alpha=31.73,$ $\beta=742.27$ | 0.041 | - | | |
| Disabling or major stroke | Beta | $\alpha=32.51,$ $\beta=741.49$ | 0.042 | - | | |
| Rehospitalisation | Beta | $\alpha=50.31,$ $\beta=723.69$ | 0.065 | - | | |
| MI | Beta | $\alpha=13.93,$ $\beta=760.07$ | 0.018 | - | | |
| Major vascular complication | Beta | $\alpha=30.19,$ $\beta=743.81$ | 0.039 | - | | |
| Life-threatening, disabling or major bleeding | Beta | $\alpha=320.44,$ $\beta=453.56$ | 0.414 | - | | |
| AKI | Beta | $\alpha=23.22, \beta=750.78$ | 0.03 | - | | |
| New PPI | Beta | $\alpha=54.95,$ $\beta=719.05$ | 0.071 | - | | |
| TIA | Beta | $\alpha=2.32,$ $\beta=771.68$ | 0.003 | - | | |
| Atrial fibrillation | Beta | $\alpha=206.66,$ $\beta=567.34$ | 0.267 | - | | |
| Paravalvular aortic regurgitation | Beta | $\alpha=3.74,$ $\beta=752.26$ | 0.0049 | - | | |
| **Utility weights in TAVI** | | | | | | |
| Month-1 | Beta | $\alpha=2289.53,$ $\beta=544.05$ | 0.81 | 0.79 | | 0.82 |
| Month-12 | Beta | $\alpha=2216.57,$ $\beta=575.08$ | 0.79 | 0.78 | | 0.81 |
| Month-24 | Beta | $\alpha=2019.54,$ $\beta=579.61$ | 0.78 | 0.76 | | 0.79 |
| **Utility weights in SAVR** | | | | | | |
| Month-1 | Beta | $\alpha=2033.39,$ $\beta=759.73$ | 0.73 | 0.71 | | 0.74 |
| Month-12 | Beta | $\alpha=1620.61,$ $\beta=415.33$ | 0.8 | 0.78 | | 0.81 |
| Month-24 | Beta | $\alpha=1537.8,$ $\beta=467.15$ | 0.77 | 0.75 | | 0.79 |

Abbreviations: AKI, acute kidney injury; CI, confidence interval; MI, myocardial infarction; PPI, permanent pacemaker implantation; SAVR, surgical aortic valve replacement; SD, standard deviation; TAVI, transcatheter aortic valve implantation
