# Supplementary material for: Cost-effectiveness of transcatheter aortic valve implantation in patients with severe symptomatic aortic stenosis of intermediate surgical risk in Singapore
Source: BMC Health Serv Res. 2022 Aug 4;22:994. doi: 10.1186/s12913-022-08369-5 (PMC9354430; doi:10.1186/s12913-022-08369-5)
Supplement: Supplementary file 4 — Additional file 4:Table S4-1. Summary of clinical outcomes used in Scenario 1 (based on PARTNER S3i PSM). Table S4-2. Utility values and decrements applied in scenario analyses. Table S4-3. Summary of clinical outcomes used in Scenario 2 (based on SURTAVI trial). Table S4-4. Results of scenario analyses. [file 12913_2022_8369_MOESM4_ESM.docx]

**Additional materials file 4**

**Scenario analyses**

The four scenario analyses were:

- Scenario 1: Clinical outcomes using SAPIEN 3 valve (PARTNER S3i/PARTNER 2A PSM)
- Scenario 2: Clinical outcomes using CoreValve® System (SURTAVI trial)
- Scenario 3: Lifetime horizon of 20 years applied on base case setting
- Scenario 4: Incorporating disutilities associated with AEs in base case

In scenario 1, clinical inputs were derived from the 1-year PARTNER S3i PSM study (Table S4-1) and modelled over a five-year time horizon. Patients in the TAVI group received the newer SAPIEN 3 valve. Monthly risks in the second year were derived by linearly projecting Month-2 and Month-12 outcomes with constant hazard rate. Beyond two years, patients in both TAVI and SAVR were assumed to follow the second-year projected probability in the SAVR arm. Table S4-2 part A shows the EQ-5D utilities from PARTNER S3i PSM [50].

In scenario 2, clinical inputs were derived from SURTAVI trial and health state utilities from PARTNER 2A (pooled TAVI (transfemoral + transthoracic)) were used (see Table S4-2 and Table S4-3 Part C). Utility decrements are the same as Scenario 4 (Table S4-3 Part B).

For scenario 3, a lifetime horizon of 20 years was applied on base case setting (PARTNER 2A inputs). The following assumptions were used:

- No valve re-intervention was required beyond five years;
- Risks were projected based on either:
  - Last observed (five-year) data in each arm; or
  - Patients in both arms follow the probabilities projected linearly based on fifth-year probability in SAVR arm.

Finally, scenario 4 evaluated the impact of AE disutilities on the cost-effectiveness of TAVI using PARTNER 2A inputs (base case). It was assumed that disabling or major stroke would incur long-term impact on utility applied in the “stroke” health state. In contrast, the occurrence of other AEs would contribute to a one-time utility decrement only. Utility decrements associated with AEs (Table S4-3 part B) were mainly obtained from Kaier et al. (2016) [51] which analysed the impact of clinical complications on QoL in high surgical risk patients aged above 75 years who received TAVI or SAVR using EQ-5D questionnaire. When utility decrement for an adverse event was not available from Kaier et al (2016) [51], decrement values from another QoL study using a Germany TAVI registry [52] and other published cost-effectiveness analyses studies on TAVI were used [17,34].

Table S4-1. Summary of clinical outcomes used in Scenario 1 (based on PARTNER S3i PSM) [13]

| **Clinical outcomes** | **TAVI (%)** | | **SAVR (%)** | |
| --- | --- | --- | --- | --- |
|  | **30 day** | **1 year** | **30 day** | **1 year** |
| All-cause mortality | 1.1 | 6.5 | 3.9 | 12.2 |
| Disabling or major stroke | 0.7 | 1.7 | 4.2 | 6 |
| Rehospitalisation | 4 | 10.5 | 6.7 | 15.2 |
| MI | 0.3 | 1.9 | 1.8 | 2.8 |
| Major vascular complication | 6.4 | - | 4.2 | - |
| Life-threatening, disabling or major bleeding | 3.6 | - | 44.2 | - |
| Endocarditis | 0.2 | 0.8 | 0 | 0.9 |
| AKI | 0.5 | - | 3.3 | - |
| New PPI | 10.5 | 12.7 | 7.6 | 10 |
| TIA | 0.4 | 1.9 | 0.3 | 1.8 |
| Atrial fibrillation | 3.2 | 4.2 | 28.5 | 29.4 |
| Paravalvular aortic regurgitation | 3.8 | 1.5 | 0.49 | 0.49 |

Abbreviations: AKI, acute kidney injury; CI, confidence interval; MI, myocardial infarction; PPI, permanent pacemaker implantation; PSM, propensity-score matched study; SAVR, surgical aortic valve replacement; TIA, transient ischemic attack; TAVI, transcatheter aortic valve implantation

Notes:

1. All-cause mortality was extracted from Kaplan-Meier curves for as-treated population, transfemoral population presented in Thourani et al., 2016 [13].
2. As paravalvular aortic regurgitation rates were not available for SAVR patients, data reported in PARTNER 2A was used instead.

Table S4-2. Utility values and decrements applied in scenario analyses

| 1. **EQ-5D utility values applied in Scenario 1** | | | | |
| --- | --- | --- | --- | --- |
| **Time point** | **TAVI, mean ± SD (95% CI)** | **SAVR, mean ± SD (95% CI)** | | **Source** |
| Baseline | 0.75 ± 0.16 | 0.74 ± 0.17 | | [50] (Utility weights were obtained from pooled [transfemoral and non-transfemoral] population) |
| Month-1 | 0.82 (0.8 to 0.83) | 0.74 (0.72 to 0.75) | |  |
| Month-12 | 0.80 (0.79 to 0.81) | 0.80 (0.79 to 0.82) | |  |
| 1. **Utility decrements associated with AEs, applied in Scenario 3** | | | | |
| **AEs** | **Utility weights, mean ± SD (95% CI)** | | | **Source** |
| Disabling or major stroke | 0.161 ± 0.537 | | | [51] |
| Rehospitalisation | 0.1167 | | | [17] |
| MI | 0.051 | | | [34] |
| Major vascular complication | 0.00695 (0.003 to 0.012) | | | [51] |
| Life-threatening, disabling, or major bleeding | 0.0463 ± 0.0154 | | | [51] |
| Endocarditis | 0.0289 | | | [17] |
| AKI | 0.177 ± 0.059 | | | [51] |
| New PPI | 0.003 ± 0.021 | | | [52] |
| TIA | 0.033 | | | [17] |
| Atrial fibrillation | 0.0377 (0.018 to 0.068) | | | [51] |
| Paravalvular aortic regurgitation | 0.024 (0.012 to 0.038) | | | [34] |
| 1. **EQ-5D utility values from PARTNER 2A (pooled TF and TT), applied in Scenario 4** | | | | |
| **Time point** | **TAVI, mean ± SD (95% CI)** | | **SAVR, mean ± SD (95% CI)** | **Source** |
| Baseline | 0.75 ± 0.16 | | 0.74 ± 0.17 | [50] |
| Month-1 | 0.82 (0.8 to 0.83) | | 0.74 (0.72 to 0.75) |  |
| Month-12 | 0.8 (0.79 to 0.81) | | 0.8 (0.79 to 0.82) |  |

Abbreviations: AKI, acute kidney injury; CI, confidence interval; MI, myocardial infarction, PPI, permanent pacemaker insertion; SAVR, surgical aortic valve replacement; SD, standard deviation; TAVI, transcatheter aortic valve implantation; TIA, transient ischemic attack; TF, transfemoral; TT, transthoracic

**Table S4-3. Summary of clinical outcomes used in Scenario 4 (based on SURTAVI trial) [12]**

| **Clinical outcomes** | **TAVI (%)** | | **SAVR (%)** | |
| --- | --- | --- | --- | --- |
|  | **30 day** | **1 year** | **30 day** | **1 year** |
| All-cause mortality | 2.2 | 6.7 | 1.7 | 6.8 |
| Disabling or major stroke | 1.2 | 2.2 | 2.5 | 3.6 |
| Rehospitalisation | 2.9 | 8.5 | 4.2 | 7.6 |
| MI | 0.9 | 2.0 | 1.0 | 1.6 |
| Major vascular complication | 6 | - | 1.1 | - |
| Life-threatening, disabling or major bleeding | 12.2 | - | 9.3 | - |
| Endocarditis | 0 |  | 0 |  |
| AKI | 1.7 | - | 4.4 | - |
| New PPI | 25.9 |  | 6.6 |  |
| TIA | 1.5 | 3.2 | 1.1 | 2.0 |
| Atrial fibrillation | 12.9 |  | 43.4 |  |
| Paravalvular aortic regurgitation | 3.5 | 1.5 | 0.7 | 0.49 |

Abbreviations: AKI, acute kidney injury; CI, confidence interval; MI, myocardial infarction; PPI, permanent pacemaker implantation; SAVR, surgical aortic valve replacement; TIA, transient ischemic attack; TAVI, transcatheter aortic valve implantation

Table S4-4. Results of scenario analyses

| Scenario | Treatment | Total costs, 2020 US$ | Total QALYs | ICER (cost per QALY), 2020 US$ |
| --- | --- | --- | --- | --- |
| Base-case | TAVI | 70959 | 2.92 | 315760 |
|  | SAVR | 39492 | 2.82 |  |
| Scenario 1: Clinical outcomes using SAPIEN 3 valve (PARTNER S3i/PARTNER 2A PSM) | TAVI | 66139 | 3.12 | 86337 |
|  | SAVR | 41,007 | 2.82 |  |
| Scenario 2: Clinical outcomes using CoreValve® System (SURTAVI) | TAVI | 67485 | 3.15 | 837595 |
|  | SAVR | 33981 | 3.11 |  |
| Scenario 3: 20-year time horizon projected based on last observed data in each arm | TAVI | 79472 | 4.85 | Dominated |
|  | SAVR | 44180 | 5.16 |  |
| Scenario 4: Incorporating disutilities associated with AEs in base case | TAVI | 70959 | 2.89 | 300070 |
|  | SAVR | 39492 | 2.78 |  |

Abbreviations: ICER, incremental cost-effectiveness ratio; PSM, propensity-score matched study; SAVR, surgical aortic valve replacement; TAVI, transcatheter aortic valve implantation
