# Supplementary material for: Cost-effectiveness of transcatheter aortic valve implantation in patients with severe symptomatic aortic stenosis of intermediate surgical risk in Singapore
Source: BMC Health Serv Res. 2022 Aug 4;22:994. doi: 10.1186/s12913-022-08369-5 (PMC9354430; doi:10.1186/s12913-022-08369-5)
Supplement: Supplementary file 5 — Additional file 5. Comparison of current model with local published 2020 study [9]. [file 12913_2022_8369_MOESM5_ESM.docx]

**Additional materials file 5**

**Comparison of current model with local published 2020 study** [9]

**Lower reported ICER despite using five-year follow-up PARTNER 2A trial data**

When compared with another published cost-effectiveness analysis (CEA) study on TAVI in intermediate risk study in Singapore , results from our study were much higher than their reported ICER of US$25,631 per QALY gained over a five-year time horizon despite also using five-year follow-up PARTNER 2A trial data [9]. These differences could be attributed to differences in model structure, health utilities, and cost differential between TAVI and SAVR.

**Model structure and health utilities**

In terms of model structure and health utilities, our study had used only stroke health states and other AEs were applied only as cost parameters since published RCT data on TAVI was unable to support mutually exclusive health states for different AEs. This is different from the use of health states for stroke, MI, and AKI in the published 2020 study from Singapore and there was insufficient information in the paper to discuss whether such AEs could be accounted for more than once [9]. The published 2020 study assumed a long-term disutility of 0.24 for stroke which was larger than the disutility of 0.161 used in the scenario analyses in our study, and ongoing AKI disutility which was applied as a one-off occurrence in our study.

**Differences in cost differential used between our model and local study**

The cost differential between TAVI and SAVR using cost extracted from Casemix and Subvention data was US$31,467. This was higher than US$13,962 in the published 2020 study from Singapore [9] which could have potentially double counted the facility fees of TAVI and SAVR; without double counting, the cost differential would be higher at US$20,428. In the absence of model validation data, it was difficult to determine the validity of the model used in the published 2020 study from Singapore [9].

**Differences in results following extended time horizon in scenario analyses**

In our study, the extrapolated trajectory for all-cause mortality beyond five years from PARTNER 2A suggests that TAVI would be worse off than SAVR. This is reflected in our scenario analyses which showed that TAVI was dominated by SAVR when the time horizon increased from five to 20 years as the simulated mortality in TAVI exceeded SAVR from 3.75 years onwards. In contrast, the published 2020 study from Singapore found that ICER reduced from US$46,080 per QALY gained to US$25,631 per QALY gained when time horizon increased from five to eight years [9].
